# Effect of Braiding Architectures on the Mechanical and Failure Behavior of 3D Braided Composites: Experimental Investigation

**DOI:** 10.3390/polym14091916

**Published:** 2022-05-08

**Authors:** Di Zhang, Xitao Zheng, Jin Zhou, Xinyi Song, Pu Jia, Haibao Liu, Xiaochuan Liu

**Affiliations:** 1 School of Mechanical, Engineering, Xi’an Jiaotong University, Xi’an 710049, China; zhangdi0719@xjtu.edu.cn (D.Z.); 3120101086@stu.xjtu.edu.cn (X.S.); p.jia6@xjtu.edu.cn (P.J.); liuxiaochuan2020@xjtu.edu.cn (X.L.); 2 School of Aeronautics, Northwestern Polytechnical University, Xi’an 710072, China; 3 Department of Mechanical Engineering, Imperial College London, London SW7 2AZ, UK; haibao.liu@cranfield.ac.uk

**Keywords:** 3D braided composite, braiding architecture, braiding angle, mechanical behavior, failure mode

## Abstract

Benefiting from the multi-directional load-bearing capability, the three-dimensional braided composites (3DBC) have found a wide application in primary structures. It is therefore of great importance to fully understand their mechanical behavior and failure modes. In the present paper, the tensile and compressive tests were carried out, according to standardized testing methods, for eight types of 3DBC, which were manufactured by resin transfer molding (RTM). It was found that the mechanical properties of the 3DBCs decreased with an increasing braiding angle. When the braiding angle was 20°, 3D 5-directional braided composite (3D5dBC) exhibited the best mechanical properties, while for the braiding angle of 40°, the mechanical properties of 3D6dBC were the most prominent. Moreover, the tensile strength of the 3DBCs is approximately two times as much as the compressive strength; however, the compressive modulus is always 10% higher than the tensile modulus. The failure modes of the 3DBCs with a braiding angle of 20°greatly depended on the braiding structures. However, they tend to be consistent when the braiding angle increases to 40°.

## 1. Introduction

Due to their outstanding structural integrity, three-dimensional braided composites (3DBCs) present excellent performances in mechanical strength and stiffness, energy absorption, damage toleration and impact resistance, compared to two-dimensional (2D) laminated composites and two and half-dimensional (2.5D) woven composites [1,2,3,4,5,6]. Thus, 3DBCs have been increasingly used in primary load-bearing structures to improve structural performance and reduce structural weight [7,8,9,10,11,12]. Given the flexible and complex spatial braiding architecture of 3DBCs, it is necessary to fully understand the effect of braiding architecture on its mechanical and failure behaviors.

In recent decades, great efforts have been focused on analytical predictive models, such as fiber inclination model [13] and the three-cell model [14], which are widely used to evaluate the elastic properties of 3DBCs. Furthermore, Chen et al. [15], Zheng et al. [16] and Mahmood et al. [17] accounted the effect of the yarn cross-section shape on the elastic properties into their predictive models. However, the analytical models are not able to precisely predict the mechanical properties and failure modes due to the over-simplified assumptions [18].

Benefiting from the finite element analysis (FEA) techniques, numerous FE models have been proposed to give an insight into the mechanical behavior and damage mechanism of 3DBCs [19,20]. Fang et al. [21,22,23] investigated the mechanical and failure performance of a 3DBC using different numerical models. Wang et al. [24] evaluated the effect of yarn deformation on the mechanical properties of a 3DBC. Gu et al. [25,26] built up full-scale microstructural numerical models to study the mechanical properties of a 3DBC. Numerical modeling techniques for the 3DBC were summarized in [27,28].

Experimental studies have been conducted to understand the mechanical and failure behaviors of 3DBCs. Filatovs et al. [29] identified the failure mode of a 3DBC by using the mode I-type notched compact tension specimens. Kalidindi and Abusafieh [30] studied the longitudinal and transverse compressive properties of a 3DBC with different braiding angles. Li et al. [31] found that the application of the cut-edge could significantly reduce the mechanical behavior and alter the failure mode of a 3DBC. Li et al. [32] introduced a novel method to test and predict the modulus of a 3DBC. A comparative study on the tensile properties between a biaxial and triaxial 3DBC has been performed by Boris et al. [33]. Recently, acoustic emission (AE) and micro-computed tomography (micro-CT) technologies have been adopted to track the damage of 3DBCs [34,35,36,37,38]. Additionally, the mechanical behaviors of 3DBCs subjected to three-point bending [39], impact [10,40,41,42] and fatigue [39,43,44] load have also been experimentally investigated.

These research endeavors promoted the application of 3DBC in engineering; however, a major defect of these analytical and numerical models is that they are valid for one braiding architecture and may run ineffective for other kinds of 3DBC. Therefore, an experimental study is still the most important method for the comprehension of the mechanical properties of 3DBC. With respect to the insufficient experimental research for 3DBC, especially for 3D six-directional (3D6d) and 3D seven-directional (3D7d) braided composites, it is still worthy of attention on the effects of braiding architectures on the mechanical behaviors, as well as the failure mechanisms. 

In the present study, the quasi-static tensile and compressive behaviors of 3DBCs with eight different braiding architectures were studied experimentally. The effects of braiding architectures on the modulus and strengths of the 3DBCs were subsequently investigated. In addition, the failure mechanism and failure modes were analyzed on the macroscopic level. The main innovation of this work is to reveal the mechanisms of the braiding structure and braiding angle of 3DBCs on the mechanical properties and failure processes, the contribution of which would benefit the industrial safety of 3DBCs as well as promote the application of 3DBCs to prominent structures. Moreover, the experimental results could provide a basis for the numerical analysis of 3DBCs.

## 2. Braiding Process and Characterization of the 3DBC 

**Figure 1:**
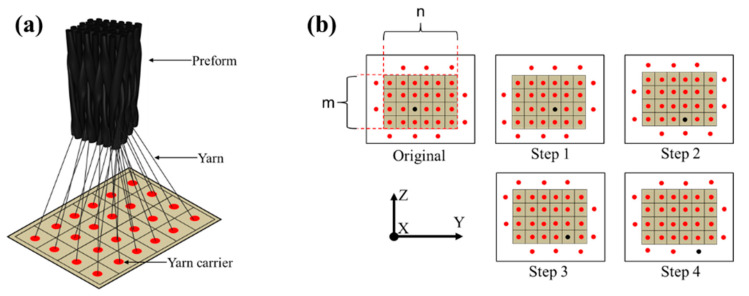
The schematic of: (**a**) the sketch map and (**b**) the yarn carrier movement in the 4 × 4 braiding process.

**Figure 2:**
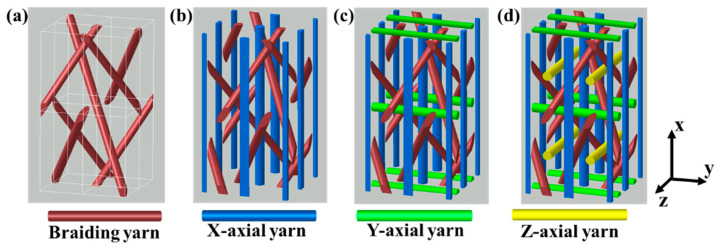
Meso-structures of (**a**) 3D4d, (**b**) 3D5d, (**c**) 3D6d and (**d**) 3D7d braided composites. Reproduced with permission from [10]. Copyright 2020 Elsevier.

**Figure 3:**
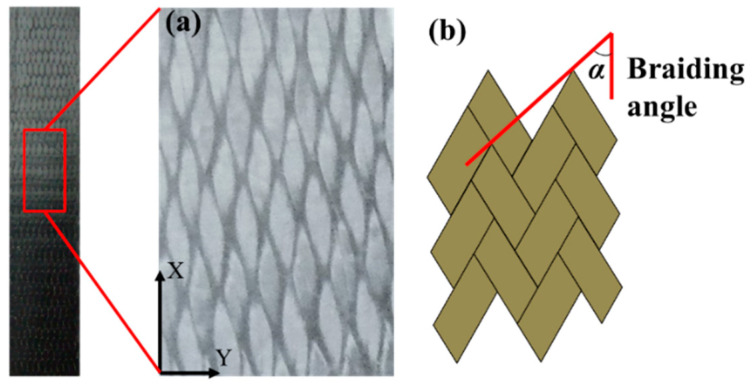
(**a**) The surface of the braided composite and (**b**) the applied braiding angle.

In the present study, the preforms of 3DBC are fabricated by the 4 × 4 braiding process, as illustrated in Figure 1. In order to study the effects of braiding architecture on the behavior of 3DBC, four braiding structures were fabricated and studied in the present paper, namely 3D four-directional (3D4d), 3D five-directional (3D5d), 3D six-directional (3D6d) and 3D seven-directional (3D7d) braiding structures, as illustrated in Figure 2, and two braiding angles of 20° and 40° were applied to describe the inclination of the braiding yarns in 3D space, as shown in Figure 3.

## 3. Specimens and Tests

**Figure 4:**
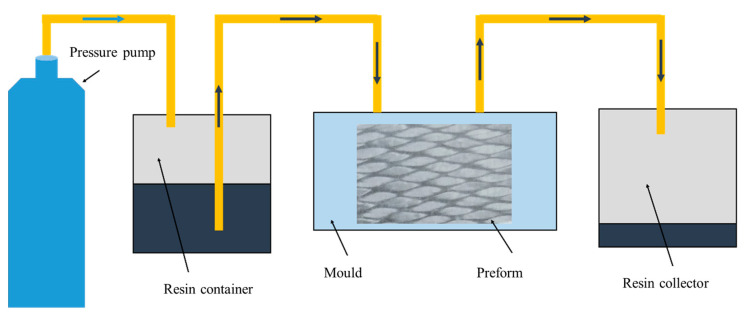
Diagram of the RTM process.

**Figure 5:**
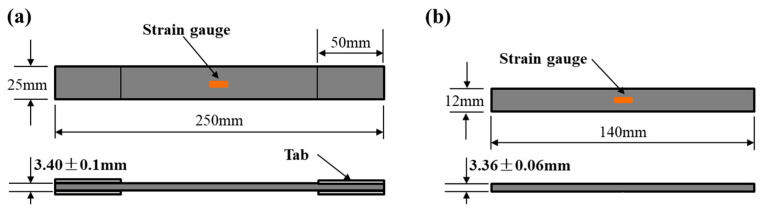
Sketch with the dimension for (**a**) tensile and (**b**) compressive specimens.

In this paper, the preformed 3DBCs were fabricated using carbon fiber tows (T700-3K, Toray^®^, Tokyo, Japan) and the epoxy resin (TDE-86, Jingdong^®^, Tianjin, China) through the resin transfer modeling (RTM) processes, as shown in Figure 4, and the mechanical properties of the fabricated 3DBC specimens are shown in Table 1. The braiding parameters, thickness and fiber volume fraction (FVF) of the specimens are listed in Table 2, and the overall dimensions of the specimens are shown in Figure 5. It should be noted that the 3DBC specimens were not edge-trimmed.

The tensile and compressive tests were conducted on a DNS200 electromechanical testing machine with a displacement control of 1 mm/min, following the ASTM D3039 [45] and ASTM D6641 [46] standards, respectively. Strain gauges were attached to specimens to record their strain response, as shown in Figure 5. Each test was repeated 5 times, and the average values were used as the results to ensure accuracy.

## 4. Results and Discussion

### 4.1. Mechanical Behavior

**Figure 6:**
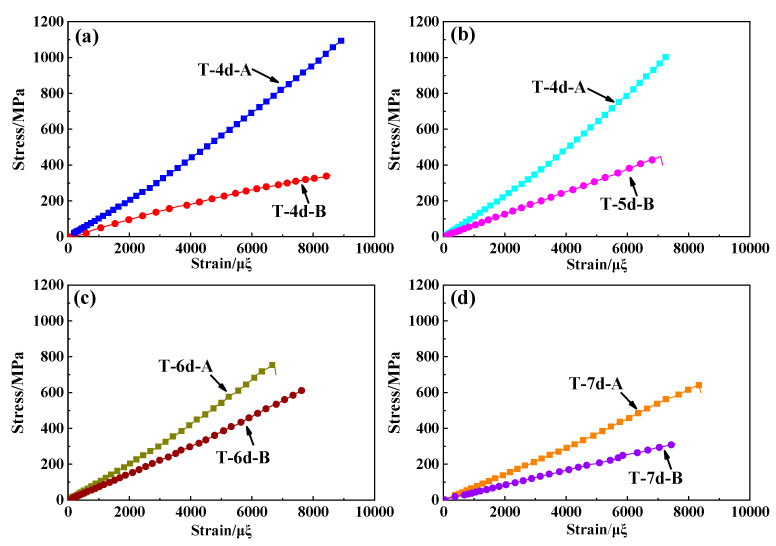
The tensile stress–strain curves of (**a**) 3D4dBC, (**b**) 3D5dBC, (**c**) 3D6dBC and (**d**) 3D7dBC.

**Figure 7:**
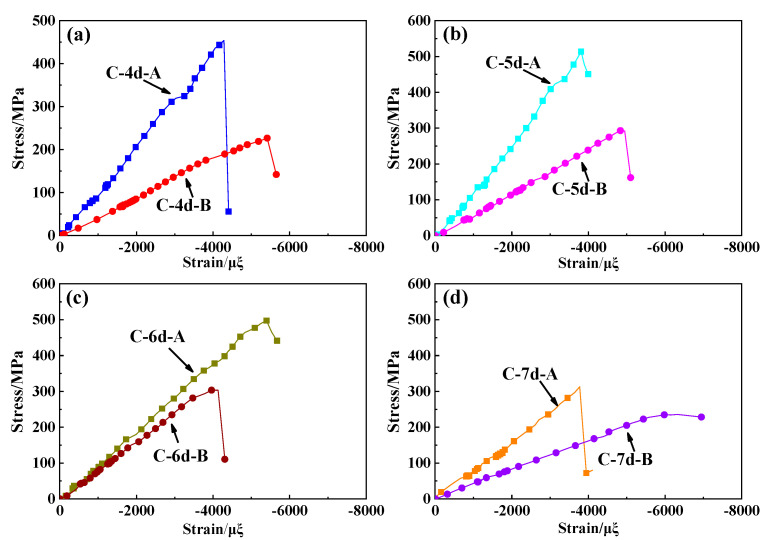
The compressive stress–strain curves of (**a**) 3D4dBC, (**b**) 3D5dBC, (**c**) 3D6dBC and (**d**) 3D7dBC.

All the stress–strain curves obtained from the tensile and compressive tests performed on the different types of 3DBC specimens tend to exhibit linear increasing trends, as presented in Figure 6 and Figure 7, respectively. It was found that the tensile/compressive modulus and strength decreased with increasing braiding angle when the same braiding structure was employed. This is because the smaller the braiding angle, the more fiber content equivalent to the longitudinal direction. Moreover, a smaller braiding angle resulted in a higher tensile failure strain and a lower compressive failure strain for the 3DBC with the same braiding structure, except for the 6d braided composite. When subjected to a tensile load, the matrix cracking and interface debonding occurred at a larger braiding angle due to the Poisson effect, thus resulting in a lower failure strain. When subjected to a compressive load, not only the matrix cracking and interface debonding but also the fiber kinking occurred at a smaller braiding angle, thus leading to a lower failure strain. Similar results were reported in [47]. As for 3D6dBC with a large braiding angle under the tensile loading conditions and a smaller braiding angle under the compressive loading conditions, it tends to inhibit the crack propagation along the longitudinal direction of the material by reducing its Poisson ratio. This phenomenon could also be found in [48], which indicated that the braiding architecture has significant effects on the mechanical behaviors of the 3DBCs. 

**Figure 8:**
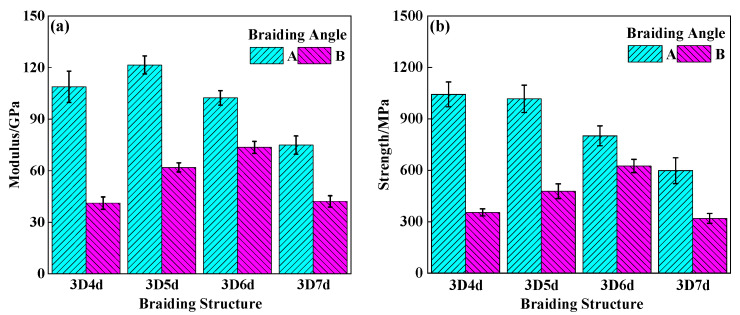
The tensile (**a**) modulus and (**b**) strength of 3DBC.

**Figure 9:**
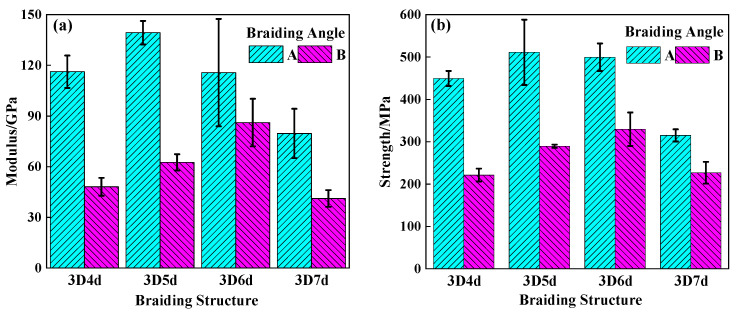
The compressive (**a**) modulus and (**b**) strength of different specimens.

Figure 8 and Figure 9 compared the tensile and compressive properties of different 3DBCs. The comparison between the results obtained from the 20° braiding composites and 40° braiding composites showed that a smaller braiding angle could result in a higher load carrying capability for the 3DBCs with the same braiding structure. In general, the tensile strength of the 3DBC should be much higher than its compressive strength. For example, the tensile strength of the T-4d-A was 2.3 times as much as its compressive strength. However, the compressive modulus of the 3DBC, except the 7d-B, was higher than its tensile modulus. Similar results were reported in [31]. This was because the dominant load-bearing component was the fiber bundle under the tensile loading conditions. When subjected to a compressive load, the support of the matrix prevented the fiber bundle from kinking, and the transverse crack propagation through the matrix was obstructed by the fiber bundles, which are much tougher and stronger, thus resulting in a higher compressive module before failure.

**Figure 10:**
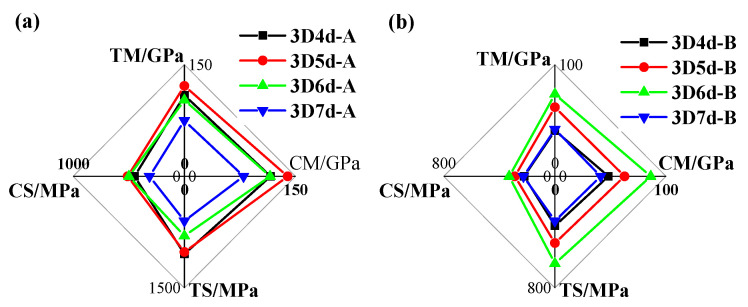
The mechanical properties of 3DBCs with the braiding angle of (**a**) 20° and (**b**) 40°. (TM, CM, TS and CS stand for tensile module, compressive module, tensile strength and compressive strength, respectively).

The mechanical properties of 3DBCs with the same braiding angle were compared in Figure 10. When using a braiding angle of 20°, the 5d-A exhibited the highest tensile and compressive strengths, among all these 20° braiding composite specimens, which were approximately 60% higher than those of the 7d-A. When using a braiding angle of 40°, the tensile and compressive strengths, as well as tensile and compressive modulus of the 6d-B, were the highest, while those values of the 4d-B and 7d-B were the lowest. The strength and modulus of the T-6d-B were 50% higher than those of the 4d-B and 7d-B. The comparison between the moduli and strengths of the 5d-A specimens and the 6d-B specimens, which are the best candidates in their corresponding group, shows that the 5d-A specimen presented the best performance.

### 4.2. Failure Modes

In order to study the effect of braiding architecture on the failure modes, the fracture characteristics of the 3DBC were analyzed from a macroscopic view. 

**Figure 11:**
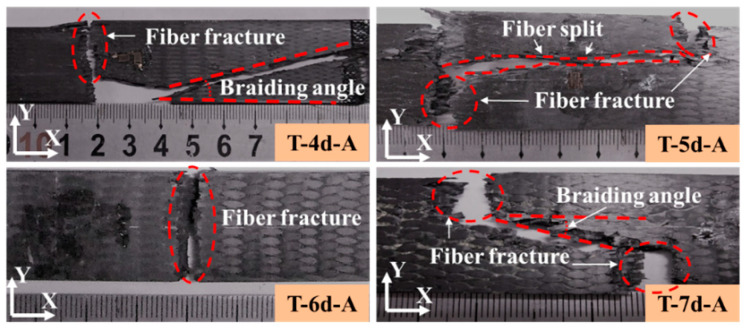
Tensile failure of the 3DBC with a braiding angle of 20°.

**Figure 12:**
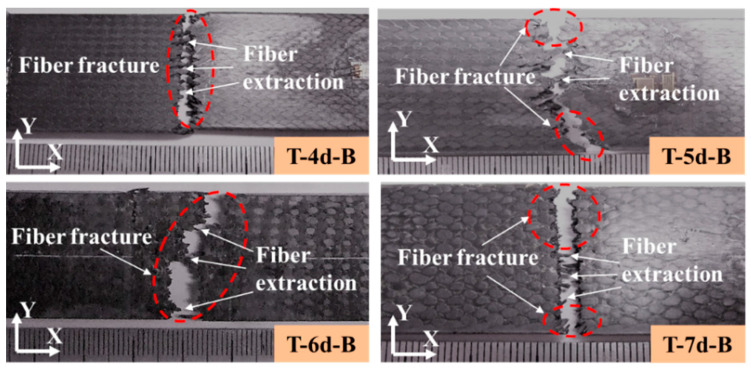
Tensile failure of 3DBC with a braiding angle of 40°.

Figure 11 and Figure 12 illustrate the tensile failure modes of the 3DBC with different braiding structures and angles. For the 3DBCs with a braiding angle of 20°, the dominant tensile failure mode of the T-6d-A was fiber fracture perpendicular to the loading direction, which was also observed from the tensile tests of other types of composite specimens, as shown in Figure 11. Differently, the fracture planes parallel to the Z-axis along the braiding angle were also found in the T-4d-A and T-7d-A specimens. For the T-5D-A, there is a fiber split parallel to the X-axis, which was not found in the tensile tests of other types of composite specimens. This is mainly because in the 3D4d and 3D5d braided composites, the matrix damage will propagate along the braiding yarns and the X-axial yarns, while in the 3D6d braided composites, the Y-axial yarns will prevent the crack from propagating along the longitudinal direction. In the 3D7d braided composite material, the content of Y- and Z-axial yarns is relatively low, and the braided and axial yarns play a major role. 

For the 3DBC with a braiding angle of 40°, the main tensile failure mode was fiber fracture perpendicular to the loading direction, suggesting that both braiding and axial yarns can carry the tensile load, as shown in Figure 12. However, an obvious difference in the fiber extractions was found in the middle region of the fracture surfaces of different types of composite specimens. This is mainly because with the increase in the braiding angle, the braided yarn will bear larger shear stress during the loading process, thus showing tensile shear failure. Therefore, the X- and Y-axial yarns significantly affect the failure modes of the 3DBC with a small braiding angle. By increasing the braiding angle, the fracture face tended to become flat, and the matrix cracking and debonding tended to grow along the interfaces, thus leading to the fiber extraction. 

**Figure 13:**
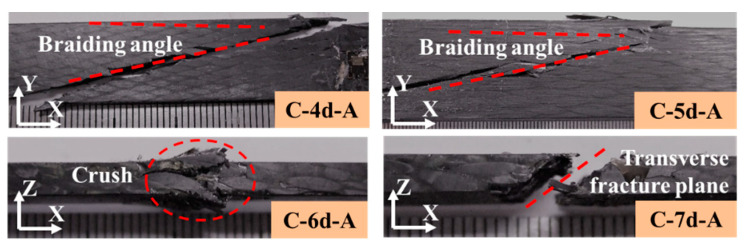
Compressive failure of 3DBC with a braiding angle of 20°.

**Figure 14:**
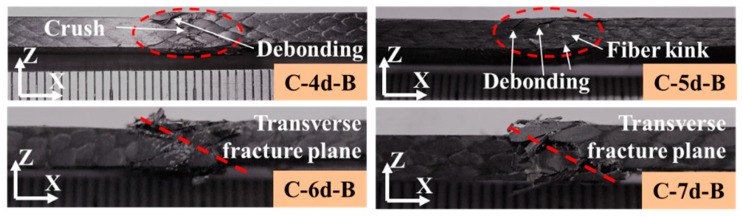
Compressive failure of 3DBC with a braiding angle of 40°.

Figure 13 and Figure 14 illustrate the compressive failure modes of the 3DBC. For the C-4d-A and C-5d-A specimens, the failure modes were compressive shear failure along the braiding angle. By increasing the braiding angle, the failure mode of the C-4d-B and C-5d-B specimens became interface debonding, resulting in sudden fiber crushing or kinking. Due to the presence of the Y-axial yarns, the compressive failure modes of the C-6d-A transferred to fiber crushing or kinking, while an obvious transverse shear fracture plane was observed from the C-7d-A specimen. For the C-6d-B and C-7d-B, the failure modes were transverse shear fracture accompanied by fiber kinking and matrix cracking. 

## 5. Conclusions

In the present paper, the effects of braiding architectures on the mechanical and failure behaviors of eight different 3DBCs were experimentally studied and analyzed. The results showed that the mechanical and failure behaviors of the 3DBC are highly related to the braiding structures and braiding angles. The influences of braiding architectures on the behaviors of the 3DBCs can be reflected by the changes in the stress–strain responses, failure strain and cracking paths, etc. Relevant conclusions have been outlined below:

(1) The modulus and strength of the 3DBC were higher with a smaller braiding angle under both tensile and compressive loading conditions. Moreover, a smaller braiding angle resulted in a higher tensile failure strain and a lower compressive failure strain of composite specimens with the same braiding structure.

(2) The 3DBC with a braiding angle of 20°, i.e., 3D5dBC, exhibited the highest tensile and compressive modulus/strengths, which were 121.48 GPa/1016.93 MPa and 139.35 GPa/511.31 MPa, respectively. In contrast, the 3DBC with a braiding angle of 40°, i.e., 3D6dBC, exhibited the highest modulus/strengths under the tensile and compressive loading conditions, which were 73.61 GPa/625.39 MPa and 84.61 GPa/329.54 MPa, respectively.

(3) With the same braiding structure and angle, the tensile strength of the 3DBC was almost two times as much as its compressive strength; however, the tensile modulus of the 3DBC was approximately 10% lower than its compressive modulus. 

(4) The dominant tensile failure modes for the 3DBC with a braiding angle of 20° were fiber splits and fracture along the braiding angle. In addition, the fiber extractions in the middle of the fracture surfaces were observed for the 3DBC with a braiding angle of 40°.

(5) By increasing the braiding angle, the compressive failure modes of the 4d and 5d braided composites transformed from the compression shear along the braiding angle to the interface debonding and fiber crushing/kinking. The dominant compressive failure modes of the 6d and 7d braided composites were transverse shear fracture regardless of the braiding angle.

## Figures and Tables

**Table 1:** Mechanical properties of T700-12K carbon fiber and TDE86 resin.

	T700-12K	TDE86
**Mechanical properties**	*E*_f1_ = 230GPa; *E*_f2_ = *E*_f3_ = 18.226 GPa; *v*_f12_ = 0.27; *v*_f13_ = *v*_f12_; *v*_f23_ = 0.3; *G*_f12_ = *G*_f13_ = 36.6GPa; *G*_f23_ = 7.01 GPa; *X*_ft_ = 4.9 GPa; *X*_fc_ = 1.4 GPa	*E*_m_ = 3.45 GPa; *v*_m_ = 0.35; *T*_m_ = 0.08 GPa; *C*_m_ = 0.180 GPa; *S*_m_ = 0.2 GPa

*E*_f1_, *E*_f2_ and *E*_f3_ represent the longitudinal, transverse and normal modulus of the fiber. *v*_f12_, *v*_f13_ and *v*_f23_ are the Poisson’s ratios of the fiber. *G*_f12_, *G*_f13_ and *G*_f23_ are the shear moduli of the fiber. *X*_ft_ and *X*_fc_ represent the longitudinal tensile and compressive strength of the fiber. *E*_m_, *v*_m_, *T*_m_, *C*_m_ and *S*_m_ represent the modulus, Poisson’s ratio, tensile strength, compressive strength and shear strength of the epoxy resin.

**Table 2:** Braiding parameters, thickness and FVF of specimens.

Specimen	m × n	Thickness(mm)	FVF (%)	Specimen	m × n	Thickness(mm)	FVF (%)
T-4d-A	6 × 34	3.29	59.7	C-4d-A	6 × 17	3.36	57.7
T-4d-B	6 × 31	3.47	59.9	C-4d-B	6 × 14	3.41	55.2
T-5d-A	5 × 29	3.50	59.7	C-5d-A	5 × 14	3.36	60.4
T-5d-B	4 × 25	3.47	53.1	C-5d-B	4 × 12	3.35	51.0
T-6d-A	4 × 25	3.48	58.3	C-6d-A	4 × 12	3.38	56.9
T-6d-B	4 × 24	3.42	55.1	C-6d-B	4 × 12	3.36	55.5
T-7d-A	4 × 25	3.42	51.2	C-7d-A	4 × 12	3.29	50.3
T-7d-B	4 × 23	3.33	52.1	C-7d-B	4 × 11	3.42	56.0

T or C corresponds to tension or compression, 4d, 5d, 6d or 7d means 3D4d, 3D5d, 3D6d or 3D7d braided composite, A or B means the braiding angle is 20° or 40°. The thickness and fiber volume fraction are the average values of five specimens. m or n represents the number of rows or columns of braiding yarns.

## Data Availability

The data presented in this study are available on request from the corresponding author.

## References

[1] Tarfaoui M., Nachtane M. (2019). Can a three-dimensional composite really provide better mechanical performance compared to two-dimensional composite under compressive loading?. J. Reinf. Plast. Compos..

[2] Zuo H.M., Li D.S., Jiang L. (2019). High Temperature Mechanical Response and Failure Analysis of 3D Five-Directional Braided Composites with Different Braiding Angles. Materials.

[3] Wu L.W., Wang W., Jiang Q., Xiang C.J., Lou C.W. (2019). Mechanical Characterization and Impact Damage Assessment of Hybrid Three-Dimensional Five-Directional Composites. Polymers.

[4] Ahmad F., Yuvaraj N., Bajpai P.K. (2020). Effect of reinforcement architecture on the macroscopic mechanical properties of fiberous polymer composites: A review. Polym. Compos..

[5] Dickson A.N., Dowling D.P. (2019). Enhancing the bearing strength of woven carbon fibre thermoplastic composites through additive manufacturing. Compos. Struct..

[6] Abbas A., Garrett W.M. (2021). Experimental evaluation of carbon fibre, fibreglass and aramid tubular braided composites under combined tension-torsion loading. Compos. Struct..

[7] Liu H., Falzon B.G., Tan W. (2018). Experimental and numerical studies on the impact response of damage-tolerant hybrid unidirectional/woven carbon-fibre reinforced composite laminates. Compos. Part B Eng..

[8] Li D.S., Yang Y., Jiang L. (2021). Experimental study on the fabrication, high-temperature properties and failure analysis of 3D seven-directional braided composites under compression. Compos. Struct..

[9] Ding G., Sun L.K., Wan Z.K., Li J.L., Pei X.Y., Tang Y.H. (2018). Recognition of Damage Modes and Hilbert-Huang Transform Analyses of 3D Braided Composites. J. Compos. Sci..

[10] Zhang D., Zheng X.T., Wang Z.B., Wu T.C., Sohail A. (2020). Effects of braiding architectures on damage resistance and damage tolerance behaviors of 3D braided composites. Compos. Struct..

[11] Liang S.Q., Zhou Q.H., Mei H.Y., Chen G., Ko F. (2020). Fatigue Behavior of 3D Braided Composites Containing an Open-Hole. Polymers.

[12] He C.W., Gao J.Y., Li H.Y., Ge J.R., Chen Y.F., Liu J.P., Fang D. (2020). A data-driven self-consistent clustering analysis for the progressive damage behavior of 3D braided composites. Compos. Struct..

[13] Yang J.M., Ma C.L., Chou T.W. (1986). Fiber Inclination Model of 3-Dimensional Textile Structural Composites. J. Compos. Mater..

[14] Wu D.L. (1996). Three-cell model and 5D braided structural composites. Compos. Sci. Technol..

[15] Chen L., Tao X.M., Choy C.L. (1999). On the microstructure of three-dimensional braided preforms. Compos. Sci. Technol..

[16] Zheng X.T., Ye T.Q. (2003). Microstructure Analysis of 4-step Three-Dimensional Braided Composite. Chin. J. Aeronaut..

[17] Shokrieh M.M., Mazloomi M.S. (2012). A new analytical model for calculation of stiffness of three-dimensional four-directional braided composites. Compos. Struct..

[18] Hallal A., Younes R., Fardoun F. (2013). Review and comparative study of analytical modeling for the elastic properties of textile composites. Compos. Part B Eng..

[19] Song S., Waas A.M., Shahwan K.W., Xiao X.R., Faruque O. (2007). Braided textile composites under compressive loads: Modeling the response, strength and degradation. Compos. Sci. Technol..

[20] Ge J.R., He C.W., Liang J., Chen Y.F., Fang D.N. (2018). A coupled elastic-plastic damage model for the mechanical behavior of three-dimensional (3D) braided composites. Compos. Sci. Technol..

[21] Fang G.D., Liang J., Wang B.L. (2009). Progressive damage and nonlinear analysis of 3D four-directional braided composites under unidirectional tension. Compos. Struct..

[22] Fang G.D., Liang J., Lu Q., Wang B.L., Wang Y. (2011). Investigation on the compressive properties of the three dimensional four-directional braided composites. Compos. Struct..

[23] Fang G.D., Wang B., Liang J. (2019). A coupled FE-FFT multiscale method for progressive damage analysis of 3D braided composite beam under bending load. Compos. Sci. Technol..

[24] Wang R.Q., Zhang L., Hu D.Y., Liu X., Cho C.D., Li B. (2017). Progressive damage simulation in 3D four-directional braided composites considering the jamming-action-induced yarn deformation. Compos. Struct..

[25] Hu M.Q., Zhang J.J., Sun B.Z., Gu B.H. (2018). Finite element modeling of multiple transverse impact damage behaviors of 3-D braided composite beams at microstructure level. Int. J. Mech. Sci..

[26] Zhou H.L., Hu D.M., Zhang W., Gu B.H., Sun B.Z. (2017). The transverse impact responses of 3-D braided composite I-beam. Compos. Part A Appl. Sci. Manuf..

[27] Gao Z.Y., Chen L. (2021). A review of multi-scale numerical modeling of three-dimensional woven fabric. Compos. Struct..

[28] Gereke T., Cherif C. (2019). A review of numerical models for 3D woven composite reinforcements. Compos. Struct..

[29] Filatovs G.J., Sadler R.L., Elshiekh A.H.M. (1994). Fracture-Behavior of a 3-D Braid Graphite-Epoxy Composite. J. Compos. Mater..

[30] Kalidindi S.R., Abusafieh A. (1996). Longitudinal and transverse moduli and strengths of low angle 3-D braided composites. J. Compos. Mater..

[31] Li J.L., Jiao Y.A., Sun Y., Wei L.M. (2007). Experimental investigation of cut-edge effect on mechanical properties of three-dimensional braided composites. Mater. Design.

[32] Li S.H., Liu L.F., Yan J.H., Yu J.Y. (2014). An approach for testing and predicting longitudinal tensile modulus of 3D braided composites. J. Reinf. Plast. Compos..

[33] Boris D., Xavier L., Damien S. (2018). The tensile behaviour of biaxial and triaxial braided fabrics. J. Ind. Text..

[34] Sikdar S., Liu D.Z., Kundu A. (2022). Acoustic emission data based deep learning approach for classification and detection of damage-sources in a composite panel. Compos. Part B Eng..

[35] John H., Silvano S., Zbigniew S., Raj D., Paul C. (2022). Digital image and volume correlation with X-ray micro-computed tomography for deformation and damage characterisation of woven fibre-reinforced composites. Compos. Struct..

[36] Liu X.D., Zhang D.T., Mao C.J., Wang X.X., Qian K. (2022). Full-field progressive fatigue damage of 3D5D braided composites with yarn-reduction: Visualization, classification, and quantification. Compos. Sci. Technol..

[37] Hao W.F., Yuan Z.R., Tang C., Zhang L., Zhao G.Q., Luo Y. (2019). Acoustic emission monitoring of damage progression in 3D braiding composite shafts during torsional tests. Compos. Struct..

[38] Zhang P.F., Zhou W., Yin H.F., Shang Y.J. (2019). Progressive damage analysis of three-dimensional braided composites under flexural load by micro-CT and acoustic emission. Compos. Struct..

[39] Zhang Z.W., Gu B.H., Sun B.Z. (2015). Experimental characterizations of three-point bending fatigue behavior of four-step three-dimensional braided composite T-beam. J. Ind. Text..

[40] Shi B.H., Liu S.K., Siddique A., Zhang J.J., Gu B.H., Sun B.Z. (2019). Comparisons on impact fracture behavior between three-dimensional four directional and five directional braided composite materials. Int. J. Damage Mech..

[41] Gao X.Z., Siddique A., Sun B.Z., Gu B.H. (2019). Effect of braiding angle on dynamic mechanical properties of 3-D braided rectangular composites under multiple impact compressions. J. Compos. Mater..

[42] Wang C., Chen Z., Silberschmidt V.V., Roy A. (2018). Damage accumulation in braided textiles-reinforced composites under repeated impacts: Experimental and numerical studies. Compos. Struct..

[43] Carvelli V., Pazmino J., Lomov S.V., Bogdanovich A.E., Mungalov D.D., Verpoest I. (2013). Quasi-static and fatigue tensile behavior of a 3D rotary braided carbon/epoxy composite. J. Compos. Mater..

[44] Ouyang Y.W., Wang H.L., Gu B.H., Sun B.Z. (2018). Experimental study on the bending fatigue behaviors of 3D five directional braided T-shaped composites. J. Text. Inst..

[45] (2014). Standard Test Method for Tensile Properties of Polymer Matrix Composite Materials..

[46] (2017). Standard Test Method for Compressive Properties of Polymer Matrix Composite Materials Using a Combined Loading Compression (CLC) Test Fixture.

[47] Wang H.L., Sun B.Z., Gu B.H. (2017). Numerical modeling on compressive behaviors of 3-D braided composites under high temperatures at microstructure level. Compos. Struct..

[48] Li D.S., Han W.F., Jiang L. (2021). On the tensile properties and failure mechanisms of 3D six-directional braided composites at elevated temperatures. Compos. Commun..

